# Effect of Warming on Personality of Mosquitofish (*Gambusia affinis*) and Medaka Fish (*Oryzias latipes*)

**DOI:** 10.3390/ani14142101

**Published:** 2024-07-18

**Authors:** Rong Wang, Baohui Yao, Zhaoxian Tan, Chengjie Mao, Yonggui Ma, Jiapeng Qu

**Affiliations:** 1 School of Life Science, Qinghai Normal University, Xining 810008, China; 18197462911@163.com (R.W.); zhaoxtan@163.com (Z.T.); 2 Sanjiangyuan Grassland Ecosystem National Observation and Research Station, Key Laboratory of Adaptation and Evolution of Plateau Biota, Northwest Institute of Plateau Biology, Chinese Academy of Sciences, Xining 810008, China; yaobaohui@nwipb.cas.cn (B.Y.); m13997138215@163.com (C.M.); 3 Key Laboratory of Medicinal Animal and Plant Resources of Qinghai-Tibetan Plateau in Qinghai Province, Xining 810008, China; 4 Academy of Plateau Science and Sustainability, People’s Government of Qinghai Province and Beijing Normal University, Xining 810016, China

**Keywords:** animal personality, climate change, behavioral syndrome, biological invasion

## Abstract

**Simple Summary:**

Temperature is a pervasive environmental factor influencing animal behavior, yet its effects on the personalities of invasive and native species remain largely unexplored. In this study, we simulated temperature increases from climate change in our lab, i.e., constant temperature treatment (control group) and warming treatment (warming group); the control group temperature was kept at 20 °C, the warming group treatment temperatures were 20 °C, 25 °C, and 30 °C. We reared mosquitofish and medaka fish under different treatments and measured personality (sociability, exploration, novelty, and boldness) in various temperature environments. The results showed that individuals of both species exhibited repeatable variation along the four behavioral axes across all temperature conditions. Sociability was significantly positively correlated with exploration, novelty, and boldness in both species, suggesting the presence of behavioral syndromes. Compared to medaka fish, mosquitofish exhibited higher dispersal ability and greater exploration in elevated temperature environments. These findings indicate that temperature is a crucial factor influencing animal personality and that the dispersal of mosquitofish may pose a potential threat to the survival of native species such as medaka fish.

**Abstract:**

Global warming may accelerate the process of biological invasions, and invasive species that can quickly adapt to new environments will have a negative impact on native species. Animal personalities have significant implications for ecology and evolution. However, few studies have simultaneously examined the combined effects of climate warming and biological invasions on native species. In this study, we hypothesized that temperature was positively correlated with personality, and invasive species had stronger personalities than native species. Accordingly, we established control (20 °C) and warming groups (20 °C, 25 °C, and 30 °C) to rear mosquitofish and medaka fish, individuals acclimatized to rearing temperatures for 7 days, then measured their personalities (sociability, exploration, novelty, and boldness). The results showed that individuals exhibited repeatable variation along the four behavioral axes across all temperature conditions, providing evidence for the presence of personalities. Significant positive correlations were found between each pair of behaviors, indicating the presence of behavioral syndrome. Sociability and exploration were most affected by temperature, showing increasing trends in sociability, exploration, and novelty in both invasive and native species with rising temperatures. Compared to medaka fish, mosquitofish exhibited higher exploration and lower sociability at elevated temperatures, while showing little change in boldness. Our results provide evidence that increased temperatures may promote biological invasions and pose a potential threat to the survival of native species. These findings are significant for understanding the complex impacts of climate change on ecosystems and for formulating effective biodiversity preservation strategies.

## 1. Introduction

Climate change poses a great challenge to global biology, primarily characterized by rising temperatures, shifting precipitation patterns, rising sea levels, etc. [1,2]. These changes have led to an increase in the frequency and intensity of extreme weather events, such as heatwaves [3,4]. According to climate change model projections, the Earth’s surface temperature will continue to rise [5], posing a significant threat to global biodiversity and ecosystem balance and jeopardizing the survival of numerous plants and animals [6,7,8,9]. For instance, climate change exposed plants to stresses such as drought, salinity, and high temperatures, which can affect their growth, development, and reproduction [10,11]. Furthermore, climate warming may encourage the reproduction and spread of pests and diseases, and large-scale outbreaks of plant diseases can lead to declines in primary productivity and biodiversity [12]. These have significant impacts on agricultural development, environmental sustainability, and socioeconomic conditions [13]. For animals, warming affects the timing of mammalian reproduction, as well as life history and morphological traits such as hibernation and body weight [14], which in turn impacts mammalian community composition [15]. Climate change also influences animal behaviors and ranges, as well as their community structure. For example, the boldness of rainbow trout (*Oncorhynchus mykiss*) changes in response to environmental challenges, suggesting that animals are continually adapting their behavior to climate change [16]. Female tiger sharks (*Galeocerdo cuvier*) migrate southward during periods of warmer temperatures, while juvenile females prefer to survive in the temperatures ranging between 22 and 23 °C [17].

In addition to these challenges, biological invasion is one of the greatest threats to biodiversity [18,19], which is the introduction of non-native species (exotic species) into new ecosystems through natural dispersal or anthropogenic activities, leading to the establishment of populations in the new environment [20]. Under the influence of climate change, invasive species with the ability to adapt quickly to their environment may further accelerate the invasion process compared to native species [21,22]. Aquatic organisms are more severely impacted by climate change than terrestrial vertebrates. The added threat from invasive species puts native species at great risk of population decline, facilitating the shift to the dominance of non-native species and providing colonization opportunities for non-native species during the process of biological invasion, and accelerates the homogenization of the global biome [8,23,24,25]. For example, the invasive guppies (*Poecilia reticulata*) outperform native twoline skiffia (*Skiffia bilineata*) in aquatic systems as temperatures rise [26]. Therefore, special attention should be given to the interactions between climate warming and invasive species in freshwater ecosystems when considering local biological conservation. Accordingly, we wanted to study the effects of warming on invasive and native species in the context of a warming climate and the effects of invasive species on native species.

In the last few decades, researchers have increasingly recognized the need to renew approaches to studying biological invasions [27], such as analyzing the successful invasion of species from the perspective of individual animals, the study of populations, and the animal personalities [28,29,30]. As has already been verified, animal personality (boldness, sociability, activity, aggression, and exploration) and associated behavioral syndromes play a crucial role in determining the likelihood of successful establishment of invasive species [31,32,33,34]. Behavioral ecologists suggest that animal behavior is plastic and animals are able to respond to changes in their surroundings (e.g., resources, predation pressures, or environment) [35]. At present, animal personalities have been proven to exist widely across animal taxa [36] and have gradually become a hot topic in animal behavior studies. Personality refers to stable, heritable behavioral differences between individual animals, formed under the combined effects of genes, acquired development, and environmental factors [37], reflecting their adaptation to specific environments, also known as “coping styles” [35,38]. This consistency in animal behavioral performance may also apply beyond a single trait to correlations between multiple traits, and this functional coupling of behavioral traits is often referred to as a behavioral syndrome [35]. For example, studies on mosquitofish have found that asocial individuals are more likely to disperse [39]. In addition, compared to native species, invasive species have higher dispersal rates, are dispersed over longer distances, or show higher reproductive rates [40,41]. Thus, sociability-dependent dispersal may be a crucial behavioral mechanism in the process of biological invasion [42]. It has been hypothesized that the successful invasion of mosquitofish was influenced by water temperature, which caused changes in the personality of mosquitofish, thereby playing a role in the invasion process [43]. Furthermore, aggressiveness is used as an indicator to assess the potential threat of invasive species. Species with high levels of aggressiveness are demonstrated to show greater competitiveness in novel environments, effectively competing with native species for re-sources and gaining an advantage in the establishment process [34,44]. In general, animals can alter their behavior to adapt to changing environments [45]. However, few studies have explored the effects of climate change on biological invasions and native species from the perspective of animal personality. Therefore, exploring the responses of both invasive and native species to climate change by simulating environmental factors under laboratory conditions can deepen our understanding of species dynamics and provide a scientific basis for assessing the risk of species invasion.

Mosquitofish (*Gambusia affinis*) is a teleost, belonging to the family Poeciliidae, characterized by ovoviviparity [46], and is native to pool and stream habitats in the southeastern and southwestern United States and northeastern Mexico [47]. It was introduced to many countries because of its positive effects in suppressing malaria epidemics and controlling mosquito populations [48] and has become the most widely distributed freshwater fish in the world [49]. Medaka fish (*Oryzias latipes*) is a small oviparous freshwater teleost native to East Asia, belonging to the family Belonidae [50]. As mosquitofish proliferate in the wild, native fishes in a similar ecological niche have suffered significant declines in abundance due to competitive effects. For example, since its introduction to Yunnan in the 1960s, the mosquitofish has competed with medaka fish, leading to the drastic decline in medaka fish, even causing its extinction in some regions [51]. The purpose of this study was to explore the effect of elevated temperature on the personality of mosquitofish and medaka fish. We raised mosquitofish and medaka fish in different temperature tanks, then measured their personality and quantified the repeatability of these traits. We further explored how elevated temperature affects personality in invasive and native species by comparing personality changes in mosquitofish and medaka fish at different temperatures. We predicted that (1) both fish would exhibit personality along four behavioral axes, i.e., repeatability, demonstrating the existence of personality; (2) sociability, exploration, novelty, and boldness were correlated (i.e., behavioral syndromes); and (3) temperature was positively correlated with personality, and compared to medaka fish, the personalities of mosquitofish varied more dramatically with elevated temperatures. This study aims to improve our understanding of invasion biology and behavioral ecology and contribute to the conservation and management of biodiversity under global climate change.

## 2. Materials and Methods

### 2.1. Experiment Animals and Rearing Conditions

The mosquitofish and medaka fish used in this study were purchased from the same commercial fish supplier (Qinghai Kate Weide Ecological Fishery Company Limited, Xining, China). Prior to the start of the trial, we used power analysis to determine the experimental animal sample size, and we selected mosquitofish (*N* = 80) and medaka fish (*N* = 80) with similar body sizes as experimental subjects and reared them in the laboratory of the Northwest Institute of Plateau Biology, Chinese Academy of Science. To investigate the effects of temperature changes on the personality of mosquitofish and medaka fish, we established two groups, i.e., control group and warming group, and set up three trials under each of the two groups. Both groups started with an initial rearing temperature of 20 °C. In the control group, the temperature was kept constant at 20 °C for the three trials, i.e., the constant temperature treatment. In the warming group, the temperatures were 20 °C, 25 °C, and 30 °C for the three trials, i.e., the warming treatment.

Upon arrival at the laboratory, mosquitofish and medaka fish were randomly assigned to either the control or warming group, with an equal number of 40 fish in each group, totaling 160 fish. The warming group of mosquitofish is represented by SH and the control group by SL. Similarly, the warming group of medaka fish is represented by QH and the control group by QL. The four groups of experimental animals were individually reared in 160 separate experimental tanks labeled with the unique identification of each fish. Each tank was a circular container (the main material is polypropylene) with a diameter of 90 mm and capacity of 300 mL, surrounded by holes to reduce water circulation between the tank and the external environment. In order to avoid mutual influence, the experimental tanks for mosquitofish and medaka fish were placed in two plastic water tanks of the same volume and size (80 cm long × 30 cm wide × 40 cm height) filled with water to a depth of 30 cm. The water in the tanks was fully filtered tap water, there were no toxic substances such as ammonia nitrogen and nitrite in the water to avoid toxic effects on fish. After supplementation with oxygen using a small oxygenating pump for 24 h, the digital PH meter was inserted into the test chamber to monitor the water quality at any time, so that the PH was maintained at 6.8–8.0. The water temperature was monitored with a digital thermometer to maintain the water temperature at 20 ± 0.5 °C. The thermometer’s sensor (usually a small probe) was placed into the water, making sure that the sensor was fully immersed, then the heater was installed in a body of water, making sure it was completely submerged. The temperature adjustment knob of the heater was set to 20 °C. The photoperiod was 12L: 12D. Mosquitofish and medaka fish were fed a floating diet with green trevally every 1 d (9:00 on the same day). Approximately 1/5 of the water in the tank was changed daily, and the bottom was promptly cleaned of food debris and feces.

All individuals were given 7 days to acclimate to the new temperature [52], after which their personalities were measured. The first behavioral measurements were per-formed on the four groups at a temperature of 20 °C. After the first measurement, the rearing temperature of the warming group was gradually increased from 20 °C to 25 °C by 0.5 °C per day [53]. The temperature of the control group was kept constant at 20 °C, and a second behavioral assay was performed on each of the four groups after the temperature of the high-temperature group reached 25 °C and the fish had acclimated for 7 days. After the second behavioral measurement, the rearing temperature of the warming group was increased from 25 °C to 30 °C as described above, while the temperature of the control group remained at 20 °C. The third behavioral measurement was conducted for each of the four groups after 7 days of warming and acclimatization. The water temperature in the chamber was monitored and recorded daily during acclimatization to ensure stability. For both groups, behavioral assays were conducted in the order of sociability, boldness, exploration, and novelty, with each individual completing the observation of one behavior before proceeding to the next.

### 2.2. Sociability Assay

**Figure 1:**
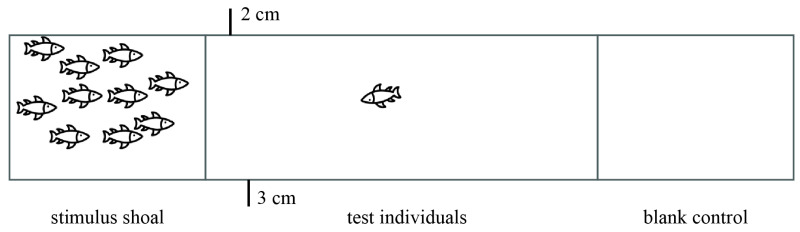
Experimental device for sociability measurement.

To quantify the sociability, we assessed individual activity in the stimulus shallows. The social assay site was an opaque white polyethylene plastic water tank (50 cm long × 20 cm wide × 30 cm height) filled with water to a depth of 14 cm. The tank was divided into three compartments (two small and one large, in a ratio of 1:2:1) using two 10 cm long transparent glass dividers (10 cm distance from the sides of the tank) for a 50 cm long tank (Figure 1). A video camera placed above the tank continuously recorded the activity of the fish. The two adjacent compartments were made of transparent glass, allowing visual interactions between the test individual and the conspecific stimulus fish group while preventing physical and olfactory interactions.

We identified the stimulus population (the stimulus shoals consisted of 10 fish of the same species, none of which had prior contact with the individuals to be tested). A stimulus shoal consisting of 10 fish was placed into one side of the compartment 1 h before the experiment, while the other side was kept blank as a control, ensuring that the stimulus shoal had no prior contact experience with the test individuals. After 1 h, the test individual was introduced to the center of the large compartment and allowed to acclimatize for 10 min. During the experiment, the tank was surrounded by black curtains, leaving a small slit for observing the fish’s activities without disturbing them. After the 10 min acclimatization period, individual behaviors were recorded for 10 min using a video camera suspended above the tank. The glass partition at the bottom of the tank was marked at 2 cm intervals, and sociability was defined as the time spent by the test individual within 2 cm of the closest stimulus fish [39]. Recorded videos were analyzed using an animal trajectory tracking system (EthoVision XT v17.5, Noldus Information Technology Co. Ltd., Wageningen, The Netherlands), and social behaviors were assessed by the following response variables: (1) distance travelled near stimulus shoal (cm), fish are more social the more distance they move near stimulated shoals; (2) percentage of time spent near stimulus shoal (%), the higher the percentage of time spent in the vicinity of stimulated shoal, the higher sociability values are.

### 2.3. Boldness Assay

To quantify the boldness, we recorded the latency of individuals to leave the shelter. Boldness was measured in an opaque white polyethylene plastic water tank (28 cm long × 18.5 cm wide × 18.5 cm height) in a well-lit environment. The water tank was filled with 10 cm of water, the tank was divided into two equal-sized areas separated by an opaque partition, and a camera was suspended above the tank. A shelter was placed on one side of the tank, which consisted of two PVC pipes with openings at the bottom of both PVC pipes. By rotating the outer PVC pipe so that the openings overlapped, the experimental target could leave the shelter, but not vice versa.

**Figure 2:**
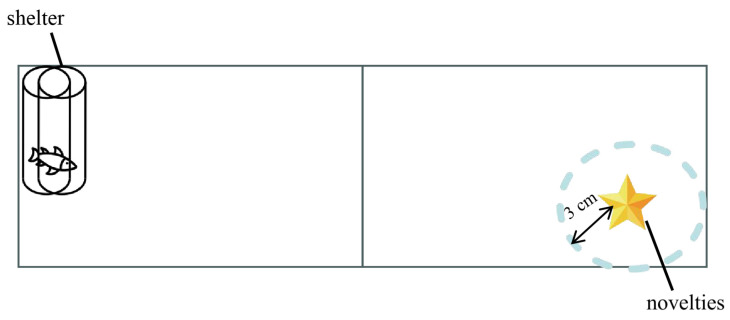
Experimental apparatus for the measurement of boldness and exploratory.

A novelty item (pentagram) was placed at the diagonal of the shelter, and a circle with a 3 cm radius was drawn around it. The time the experimental target spent within this circle was recorded as a measure of its interaction with the novelty (Figure 2). The tank was surrounded by black curtains during the experiment, with a small slit allowing us to observe the fish’s activities without disturbing them.

At the beginning of the experiment, individuals that had their sociability measured were tested for boldness. The experimental target was placed in the aforementioned shelter, and after 10 min of acclimatization, a switch on the shelter was remotely flipped, and the clear glass panel was removed to allow the fish to enter the experimental arena. The behavior of the animals was recorded using a video camera to record the time of the first sustained (≥10 s) departure of the target individual from the shelter over a 5 min period. Boldness was measured based on the latency for the target individual to leave the shelter; the longer the latency is, the lower the boldness values are. For easy understanding and computation, we transform the latency to leave the shelter into the time spent continuously outside the shelter (s); the longer the time outside the shelter, the higher the boldness values are (the time spent continuously outside the shelter was the maximum time allowed for fish to leave the shelter, 300 s, minus the latency to leave the shelter and the time spent out of the shelter for more than 10 consecutive seconds). In the results, “boldness” was expressed in terms of the time spent continuously outside the shelter.

### 2.4. Exploration Assay

In order to quantify an individual’s level of exploration, a novel environment was used for testing. This novel environment is a rectangular tank (a new opaque white polyethylene plastic water tank, not the tank that measured sociability and boldness). Immediately prior to testing, the individual’s residence (shelter) was removed from its original experimental tank and placed in the center of the new tank. The central shelter was a standardized starting location and familiar environment; individuals could choose to retreat to the shelter, thus allowing for the quantification of true exploration rather than forced exploration of novel environments. Individuals were recorded using a video camera suspended above the tanks. They were placed directly into their respective shelters for a 5 min acclimatization period. After this period, the shelter was gently removed, and individual behavior was recorded for 15 min from the moment the shelter was out of the camera’s field of view. Recorded videos were analyzed using an animal trajectory tracking system (EthoVision XT, Noldus Information Technology Co. Ltd., Holland), and exploratory behaviors were assessed by the following response variables: (1) distance travelled (cm), the longer distance is, the higher exploration values are; and (2) percentage of time spent moving (%), the higher percentage is, the higher exploration values are.

### 2.5. Novelty Assay

The novelty assay and the exploration assay were performed in the same experimental arena. At the end of the exploration test, novelties were suddenly thrown into the center of the experimental tank. The following novelties for the three experiments were used: 2 cm^3^ Lego bricks, ping pong balls filled with sand, and an irregular polyhedron. A circle with a radius of 3 cm was drawn around the novelty item. The time and behavior of the experimental target staying within this circle was recorded to assess novelty. The behavior of each individual was recorded via the downward-facing camera for a period of 10 min. Recorded videos were analyzed using an animal trajectory tracking system (EthoVision XT, Noldus Information Technology Co. Ltd., Holland), and novel behaviors were assessed by the following response variables: (1) distance travelled (cm); and (2) percentage of time spent moving (%), high values mean high novelty.

### 2.6. Ethical Statement

The experiments were conducted in strict compliance with current Chinese regulations on animal welfare and research ethics. The experimental animals were reviewed and approved by The Ethics Committee of the Northwest Institute of Plateau Biology, Chinese Academy of Sciences (NWIPB-2019018). No physically invasive manipulations were performed on the fish during the experiments and no signs of distress or struggle were observed. At the end of the experiments, all experimental fish were safely released to their original holding tanks.

### 2.7. Statistical Analyses

The experimental videos were analyzed using an animal movement trajectory tracking system (EthoVision XT, Noldus Information Technology Co. Ltd., Holland) to obtain data on four personalities of mosquitofish and medaka fish. Typically, repeatability is used as a measure of individual behavioral consistency in animal behavioral studies [54]. Here, we utilized the “*rptR*” package to measure the repeatability of sociability [55], exploration, novelty, and boldness, with trials as a fixed factor and individual ID as a random effect. To estimate the confidence intervals, the number of parametric bootstrap iterations was controlled by setting the nboot argument to 1000 bootstraps [47]; the npermut argument controls the number of randomizations for permutation-based null hypothesis testing, and in this paper, this parameter is set to 1000 [55]. In the calculations, we found that temperature variations have a large effect on the repeatability estimates, so the repeatability of mosquitofish and medaka fish was calculated separately for the high- and low-temperature groups.

We also wanted to test behavioral syndromes in mosquito fish and medaka fish. To achieve this, we utilized prior distributions and mixed-effects multivariate models from the “*MCMCglmm*” package to estimate covariances and correlations between pairs of personality and to test for the presence of behavioral syndromes. The parameters of the prior distribution were an expected variance V = diag (2) and a degree of belief nu = 1.002 [56]. Each pair of personality (sociability, exploration, novelty, and boldness) was treated as dependent variables in multivariate mixed models, and the models were run for 1,500,000 iterations with a burn-in phase of 500,000 iterations and thinning interval of 100 iterations [56]. At the end of the model run, the posterior probability distribution plots were observed using the plot function to ensure proper model mixing and convergence. Finally, within-individual, among-individual, and phenotypic variances were calculated separately. Phenotypic variance is divided into among-individual variance and within-individual variance, with the former representing behavioral syndrome and the latter representing plasticity integration, and phenotypic correlations are the result of a combination of within-individual correlations and among-individual correlations. The final results are presented as correlation estimates and 95% credible intervals, which are considered significant when the credible intervals do not overlap zero.

We used a linear mixed-effects model (LMM) to analyze the changes in the relative importance of the effects of species, group, and acclimated temperature on four animal behaviors. The model explained the proportion of species, group, acclimated temperature, and their interactions, where the main effects are the (1) species effect (i.e., mosquitofish vs. medaka), (2) treatment effect (control group vs. warming group), and (3) acclimated temperature effect (20, 25, and 30). The interaction effect is (1) between species and group treatment and (2) between species and acclimated temperature, with ID as a random effect. We used the “*lem4*” package to fit the LMM [57]. A hierarchical split for the proportion of explanations in each section was performed using the “*glmm.hp*” package [58]. Based on the results of linear mixed-effects modeling, we explored the effects of warming and control groups on the personality of two fish species. For the three behavioral traits, we used the Shapiro–Wilk test to examine whether the data conformed to a normal distribution, and since the data were not normally distributed, we conducted a Mann–Whitney U test to compare animal personalities between the high and low temperature groups [53]. All analyses were conducted in R (v. 4.4.1).

## 3. Results

### 3.1. Behavioral Repeatability Estimates

Calculating the repeatability of each personality axis, the repeatability estimates calculated for the two measures of sociability and exploration in mosquitofish and medaka fish confirmed that all two measures (distance travelled near stimulus shoal or distance travelled, percentage of time spent near stimulus shoal or percentage of time spent moving) had medium repeatability. The repeatability estimates calculated were low for the two measures of novelty (distance travelled and percentage of time spent moving) and boldness, both in the low-to-medium range. Mosquitofish reared in the control group had a repeatability ranging from 0.291 to 0.333, and the warming group had a repeatability ranging from 0.287 to 0.333 (Table 1). The repeatability ranged from 0.291 to 0.333 for medaka fish reared in the control and warming group (Table 2). The sociability, exploration, novelty, and boldness of two species showed a similar pattern of repeatability in both groups, which suggests that all measured behaviors had a medium level of repeatability over time. Overall, the repeatability of boldness was higher in the control groups than in the warming groups, and mosquitofish were more repeatable than medaka fish.

### 3.2. Behavioral Syndromes

We used distance traveled to assess sociability, exploration, and novelty and found that among-individual correlations between traits ranged from 0.01 to 0.36 in mosquitofish (Table 3) and ranged from −0.02 to 0.02 in medaka fish (Table 4). When using the percentage of time (percentage of time spent near stimulus shoal or percentage of time spent moving) to calculate correlation, the range was −0.03 to 0.23 in mosquitofish (Table S1) and −0.01 to 0.13 in medaka fish (Table S2). HPDs for all traits included zero, which indicates that the among-individual correlations were quite low and negligible (i.e., absence of correlation at the among-individual level).

At the within-individual level, we used distance traveled to assess behavior correlation and found that sociability was positively significantly correlated with exploration, novelty, and boldness in mosquitofish (Table 3) and medaka fish (Table 4). Furthermore, exploration was positively significantly correlated with novelty and boldness in medaka fish (Table 3) and mosquitofish (Table 4). A strong positive within-individual correlation between these behaviors drove the overall positive phenotypic correlation observed throughout the models. In contrast, boldness was not correlated with novelty in either fish at the among-individual, within-individual, or phenotypic levels (Table 3 and Table 4).

### 3.3. Effects of Factors on Behavioral Traits

#### 3.3.1. Sociability

**Figure 3:**
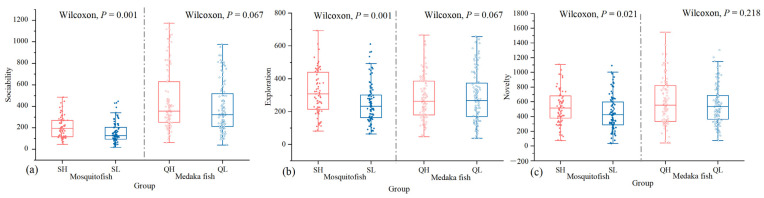
Effect of temperature group on personality of mosquitofish and medaka fish. (**a**) Sociability: distance travelled near stimulus shoal; (**b**) exploration: distance travelled; (**c**) novelty: distance travelled. Groups SH and SL represent mosquitofish exposed to warming and control groups, respectively. Groups QH and QL represent medaka fish exposed to warming and control groups, respectively.

**Figure 4:**
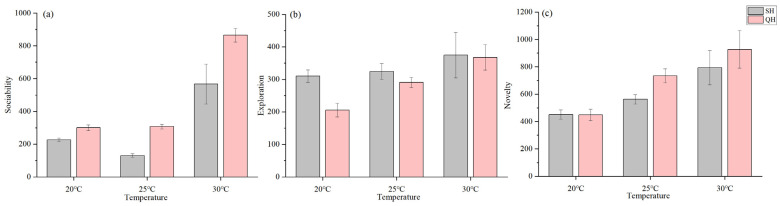
The effect of temperature changes in the warming group on the personality of animals. (**a**) Sociability: distance travelled near stimulus shoal; (**b**) exploration: distance travelled; (**c**) novelty: distance travelled. Groups SH and SL represent mosquitofish exposed to warming and control groups, respectively. Groups QH and QL represent medaka fish exposed to warming and control groups, respectively.

With regard to sociability, the fixed factors, that is, species, group, and acclimated temperature, significantly explained the differences in distance travelled near the stimulus shoal (*p* < 0.05), with a significant interaction between species and group and between species and acclimated temperature for all two measures of sociability (Table 5 and Table S3). The sociability of mosquitofish was significantly higher in the warming group than the control group (*p* = 0.001, Figure 3a); similarly, the sociability of medaka fish was higher in the warming group than in the control group, although this difference was not significant (*p* = 0.067, Figure 3a). Overall, under both groups, the sociability of medaka fish was significantly higher than that of mosquitofish (*p* < 0.05, Figure 3a). In the warming group, the distance travelled near stimulus shoal of both mosquitofish and medaka fish increased after the temperature increased (Figure 4a), and the percentage of time spent near the stimulus shoal of mosquitofish increased after the temperature increased, with an opposite trend in medaka fish (Figure S1). Medaka fish were more social than mosquitofish at all three acclimated temperatures, and their sociability was significantly higher than that of mosquitofish when the temperature increased to 30 °C (*p* = 0.001, Figure 4a and Figure S1).

#### 3.3.2. Exploration

As for exploration, we found a significant effect on acclimated temperature for all two measures of exploration, and there was also a significant interaction between species and group and between species and acclimated temperature for distance travelled (*p* < 0.05, Table 5 and Table S3). The exploration of mosquitofish was significantly higher in the warming group than control group (*p* = 0.001, Figure 3b). Similarly, the exploration of medaka fish was higher in the warming group than in the control group, but this effect was not significant (*p* = 0.895, Figure 3b). Overall, the exploration of mosquitofish in the warming group was higher than in the other three groups (Figure 3b). In the warming group, the two measures of exploration in mosquitofish and medaka fish increased with rising rearing temperature (Figure 4b and Figure S1). The magnitude of change was greater in mosquitofish than in medaka fish, when the fish were reared at 20 °C, the distance travelled and percentage of time spent moving by mosquitofish was significantly higher than medaka fish (*p* = 0.014, Figure 4b and Figure S1).

#### 3.3.3. Novelty

In terms of novelty, species, group, and acclimated temperature of individuals were significantly related to the distance travelled during boldness assays, and acclimated temperature was a significant predictor of the percentage of time spent moving (*p* < 0.05, Table 5 and Table S3). The novelty of mosquitofish was significantly higher in the warming group than control group (*p* = 0.021, Figure 3c). Similarly, the novelty of medaka fish was higher in the warming group than control group, but this difference was not significant (*p* = 0.218, Figure 3c). Overall, under both groups, the novelty of medaka fish was higher than mosquitofish (Figure 3c). In the warming group, the distance travelled in mosquitofish and medaka fish increased with rising rearing temperature (Figure 4c), when the fish were reared at 25 °C, the distance travelled by medaka fish was significantly higher than mosquitofish (*p* = 0.015, Figure 4c). The percentage of time spent moving by mosquitofish and medaka fish increased and then decreased with rising rearing temperature (Figure S1), and the percentage of time spent moving was significantly higher in mosquitofish than medaka fish at 25 °C.

#### 3.3.4. Boldness

For boldness, under both groups, the time spent continuously outside the shelter of mosquitofish and medaka fish ranged from 0 to 300 s. In the warming group, the average time for mosquitofish to be continuously outside the shelter was 186.40 s, with an average of 168.75 s in control group. In the warming group, the average time for medaka fish to be continuously outside the shelter was 197.28 s, and in the control group, it was 200.39 s. Species, group, and acclimated temperature of individuals were not significantly related to boldness (*p* > 0.05, Table 5).

## 4. Discussion

In this study, we examined the effects of temperature on two fishes’ personalities. We observed that sociability and exploration were most affected by temperature. Both mosquitofish and medaka fish exhibited upward trends in sociability and exploration as temperature increased. However, mosquitofish showed stronger exploratory and dispersal abilities than medaka fish. The sociability, exploration, and novelty of mosquitofish were significantly higher in the warming group compared to the control group. In contrast, the sociability, exploration, and novelty of medaka fish showed no significant variance between the warming and control groups, suggesting that mosquitofish are more adaptable to environment changes than medaka fish.

### 4.1. Behavioral Repeatability and Behavioral Syndromes

The repeatability of behavior is the foundation for the study of animal personality [59,60,61]. Repeatability of personality means that the behavioral traits of an animal are consistent over time or context and that behavioral differences between individuals are much larger than within-individual variations in behavioral plasticity, thus repeatability is the primary basis for determining the presence of personality in animals [60]. The presence of repeatability suggests that animals tend to maintain a consistent pattern of behavior when faced with changes in the external environment and internal physiological states. For example, zebrafish exhibited evasive behavior in the presence of potential threats, and the behavior remains highly consistent across test conditions [62]. Previous research has confirmed that over 35% of the phenotypic variance in animal populations can be attributed to individual behavioral variances, which can significantly influence the evolution and natural selection of these populations [35]. Additionally, the meta-analysis showed that the repeatability values for vertebrates ranged from 0.001 to 0.930 with a mean value of 0.380 [63]. Our results found that sociability, exploration, novelty, and boldness were moderately repeatable at all temperatures in mosquitofish and medaka fish; particularly, boldness exhibited higher repeatability in the control group compared to the warming group. This finding is consistent with previous research studies showing that mosquitofish exhibit significant behavioral repeatability at low temperatures [47]. Previous studies in wormlion larvae (*Vermileo vermileo*) have also found that animals’ behavior is more erratic and variable at higher temperatures [64]. The stability of behavior changes depending on the growing environment, suggesting that environmental temperature may be an important factor influencing personality in ectotherms [65]. At higher temperatures, animals may increase within-individual behavioral plasticity in response to environmental changes, thereby enhancing their adaptability. Additionally, mosquitofish exhibited higher repeatability in four behaviors compared to medaka fish, suggesting tha mosquitofish can minimize the costs of adjusting behavioral strategies. This adaptability aids in the expansion of their territories amid global warming conditions [66]. Moreover, these consistent individual behavioral differences provide mosquitofish with a broader ecological niche and greater environmental adaptability compared to native species, thereby enhancing ecosystem diversity.

The correlations between behaviors, reflecting how individuals cope with environmental challenges, are known as the behavioral syndrome or personality [35]. In this study, to quantify genuine behavioral syndromes, we decomposed phenotypic correlations into within-individual correlations and among-individual correlations [67]. Our results showed significant positive correlations between the behaviors of both mosquitofish and medaka fish. However, among-individual correlations were negligible compared to within-individual correlations, which suggests that within-individual correlations primarily drive the observed phenotypic correlations. Furthermore, our results indicated that asocial individuals were more adventurous, curious about novel environments, dispersed farther, and were more likely to explore new and potentially dangerous environments. These tendencies may be determined by internal factors such as hormones. Notably, behavioral traits in animals often do not evolve in isolation, and behavioral syndromes are prevalent in nature [35]. Therefore, studying the correlations of these behavioral traits can deepen our understanding of animals’ strategies for adapting to their environments.

### 4.2. Effects of Temperature on Personality

With regard to sociability, sociability is widespread in all types of populations, and it refers to the affinity or avoidance responses of individuals when confronted with others of their own species [68]. It is an important measure of dispersal distance, as asocial individuals tend to disperse farther [28]. Increased temperatures due to global warming play an important role in altering the sociability of ectotherms. As expected, the sociability of both fishes increased with rising temperatures, which is contrary to findings that high temperatures can reduce sociability in some ectotherms [69]. This can be explained by the fact that fish are more inclined to join smaller social groups [70]. Additionally, appropriate temperature increases can improve food resources, potentially reducing the dispersal distance of individuals [71]. In contrast, Briffa reported that the relationship between temperature and personality in hermit crabs (*Pagurus bernhardus*) was influenced by prior temperature acclimation [72]. It is possible that during the gradual daily warming process, individuals might adjust their sociability to adapt to the temperature changes, thereby maintaining group stability and survival [73], therefore, sociability increased at higher temperatures. Enhanced sociability means more frequent and closer cooperative behavior between individuals, which not only improves the overall survival and reproductive success of the population [74], it also reduces intraspecific competition and promotes the development of interspecific symbiotic relationships, thereby increasing ecosystem stability and diversity [64]. In addition, the overall trend of personality with increasing temperature was similar for both fishes; however, medaka fish were more social than mosquitofish at the same temperature, indicating that the dispersal distance of mosquitofish was larger than medaka fish. As expected, the invasion of mosquitofish negatively affects native species by competing for food and space resources, which is consistent with the results of many previous studies [40,42]. This is because mosquitofish are characterized by fast growth, adaptability, and high fecundity, which facilitates their dominance in ecological competition and enables them to crowd out native species, thus dispersing to a broader area [49]. This may also be a protective phenomenon for the invasive species against itself, as the risk of disease transmission usually increases as temperatures rise, and mosquitofish may reduce the risk of contracting parasites by reducing their social behavior [75]. It has been found in primate studies that the variation in the serotonin transport protein gene is associated with among-individual differences in social behavior [76], suggesting that genetic variation plays an important role in the regulation of social behavior. This provides us with ideas for subsequent mechanistic studies.

For exploratory behavior, the exploration of individuals actually reflects their metabolic rate [77]. Since the metabolic rate of animals is closely related to temperature, the 0.5 °C increments per day in this study may lead to an increase in the metabolic rate of fish [78], and an increased metabolic rate means that more energy and resources are required to maintain physiological activity. In order to compensate for the rising metabolic costs, animals will increase their exploratory and novelty behaviors to search for food resources [79,80], and this may explain why rearing temperature has a significant effect on exploration and novelty. Our results indicated that fish reared in the warming group had more exploratory and novelty behaviors than those in the control group, and this is consistent with previous results and illustrates the plasticity of behavioral variations in mosquitofish and medaka fish [52], enabling them to change their state to adapt to environ-mental change. Furthermore, as expected, mosquitofish were more exploratory than medaka fish at the same temperature, and the invasion would occupy more resources and affect the survival of medaka fish. Mosquitofish are more adaptable to new environments due to their frequent introduction for pest control, enabling them to positively explore the environment they inhabit [48]. In the context of a warming climate, geographic isolation may contribute to the formation of new species as species explore new habitats [45]. Increased exploration may also lead to the invasion of some species into new ecological regions, posing a threat to native species and affecting local species composition and ecological balance [81]. In addition, we found that mosquitofish were less social than medaka fish, which means that medaka fish prefer to live in clusters. This preference makes medaka fish reluctant to take more risks and take actions to positively explore new environments. This may be caused by internal factors such as hormones [82]. As the global climate warms, increased exploration and dispersal distances may impact species migration patterns and ranges [83] and even accelerate the invasion process.

In terms of boldness, we did not observe any effect of temperature on boldness in mosquitofish and medaka fish. This finding suggests that, to some extent, personality determines the behavioral performance of animals, meaning that they may not achieve the desired behavioral state in all situations. As the temperature changes, some individuals may exhibit behavioral variations to adapt to the environment, demonstrating plasticity, while the personalities of some animals may be relatively stable and less susceptible to changes in the external environment, demonstrating behavioral stability [35,38]. Boldness refers to an individual’s ability to respond to extrinsic risks in a familiar environment, reflecting the animal’s predation risk and survival [84]. In our study, the tested individuals were not exposed to predation risks, which explains why there was no difference in boldness. In our study, we used the time spent continuously outside the shelter as a measure of boldness, and the lower repeatability and unsatisfactory results of boldness are due to the improper choice of indicator [85].

## 5. Conclusions

Our study demonstrates how temperature changes affect the personalities of mosquitofish and medaka fish. Our study found that both mosquitofish and medaka fish exhibit personality and behavioral syndromes. As the temperature of the warming group increased, medaka fish were significantly more social than mosquitofish, suggesting that mosquitofish have greater dispersal abilities [28]. Mosquitofish were significantly more exploratory than medaka in the warming group, suggesting that higher exploration in mosquitofish may affect the migration patterns and distribution of the species and even accelerate its invasion rates [47]. These results suggest that mosquitofish are better adapted to climate change than medaka fish. However, only one indicator (temperature) corresponded to climate change in this study, and further investigations are recommended to explore the impact of multiple indices (e.g., temperature, carbon dioxide content, and oxygen content) of climate change on the personality of invasive and native species. The effective prevention and control of invasive species are strategic needs to ensure national ecological security, and these studies can provide a stronger scientific basis for local ecological conservation and management.

## Figures and Tables

**Table 1:** Repeatability estimates (*R*) and confidence intervals (CI) of sociability, exploration, novelty, and boldness behavior in *Gambusia affinis* reared in different groups.

Behavior	Response Variable	Group	*R* ± SE	CI
Sociability	distance travelled near stimulus shoal (cm)	warming group	0.333 ± 0.174	0.002, 0.591
		control group	0.333 ± 0.118	0.152, 0.597
	percentage of time spent near stimulus shoal (%)	warming group	0.332 ± 0.138	0.111, 0.630
		control group	0.332 ± 0.167	0.010, 0.578
Exploration	distance travelled (cm)	warming group	0.333 ± 0.136	0.134, 0.644
		control group	0.333 ± 0.126	0.139, 0.623
	percentage of time spent moving (%)	warming group	0.327 ± 0.145	0.107, 0.649
		control group	0.324 ± 0.128	0.139, 0.611
Novelty	distance travelled (cm)	warming group	0.333 ± 0.134	0.120, 0.643
		control group	0.333 ± 0.120	0.149, 0.607
	percentage of time spent moving (%)	warming group	0.292 ± 0.193	0.004, 0.686
		control group	0.299 ± 0.174	0.001, 0.633
Boldness	time spent continuously outside the shelter (s)	warming group	0.287 ± 0.146	0.001, 0.576
		control group	0.291 ± 0.172	0.060, 0.621

**Table 2:** Repeatability estimates (*R*) and confidence intervals (CI) of sociability, exploration, novelty, and boldness behavior in *Oryzias latipes* reared in different groups.

Behavior	Response Variable	Group	*R* ± SE	CI
Sociability	distance travelled near stimulus shoal (cm)	warming group	0.333 ± 0.112	0.173, 0.612
		control group	0.333 ± 0.108	0.178, 0.595
	percentage of time spent near stimulus shoal (%)	warming group	0.331 ± 0.115	0.161, 0.601
		control group	0.331 ± 0.158	0.176, 0.606
Exploration	distance travelled (cm)	warming group	0.333 ± 0.114	0.167, 0.595
		control group	0.333 ± 0.110	0.171, 0.593
	percentage of time spent moving (%)	warming group	0.325 ± 0.125	0.136, 0.617
		control group	0.324 ± 0.154	0.149, 0.581
Novelty	distance travelled (cm)	warming group	0.333 ± 0.110	0.177, 0.591
		control group	0.333 ± 0.109	0.179, 0.591
	percentage of time spent moving (%)	warming group	0.297 ± 0.139	0.045, 0.605
		control group	0.297 ± 0.152	0.039, 0.601
Boldness	time spent continuously outside the shelter (s)	warming group	0.291 ± 0.134	0.100, 0.620
		control group	0.291 ± 0.157	0.129, 0.588

**Table 3:** Results of testing binary correlations between sociability (distance travelled near stimulus shoal), exploration (distance travelled), novelty (distance travelled), and boldness (time spent continuously outside the shelter) behaviors with MCMC general linear mixed-effects models. The best estimates of correlation coefficients (values above the diagonal) and their 95% credibility intervals (values below the diagonal) are presented for among-individual, within-individual, and phenotypic correlations in *Gambusia affinis*. Significant results corresponding to correlation coefficients whose credibility intervals do not overlap zero are shown in bold.

		Sociability	Exploration	Novelty	Boldness
Sociability	Among-individual	-	0.05	0.05	0.01
	Within-individual	-	**0.24**	**0.25**	**0.20**
	Phenotypic	-	**0.20**	**0.21**	**0.19**
Exploration	Among-individual	−0.51, 0.56	-	0.36	0.08
	Within-individual	**0.08, 0.40**	-	**0.43**	**0.25**
	Phenotypic	**0.06, 0.33**	-	**0.42**	**0.21**
Novelty	Among-individual	−0.63, 0.68	−0.32, 0.80	-	0.21
	Within-individual	**0.08, 0.39**	**0.23, 0.59**	-	0.14
	Phenotypic	**0.07, 0.34**	**0.29, 0.55**	-	0.14
Boldness	Among-individual	−0.83, 0.87	−0.48, 0.62	−0.79, 0.89	-
	Within-individual	**0.05, 0.35**	**0.07, 0.42**	−0.05, 0.31	-
	Phenotypic	**0.05, 0.34**	**0.08, 0.36**	−0.02, 0.28	-

**Table 4:** Results of testing binary correlations between sociability (distance travelled near stimulus shoal), exploration (distance travelled), novelty (distance travelled), and boldness (time spent continuously outside the shelter) behaviors with MCMC general linear mixed-effects models. The best estimates of correlation coefficients (values above the diagonal) and their 95% credibility intervals (values below the diagonal) are presented for among-individual, within-individual, and phenotypic correlations in *Oryzias latipes*. Significant results corresponding to correlation coefficients whose confidence intervals do not overlap zero are shown in bold.

		Sociability	Exploration	Novelty	Boldness
Sociability	Among-individual	-	0.01	0.01	0.001
	Within-individual	-	**0.47**	**0.27**	**0.15**
	Phenotypic	-	**0.43**	**0.24**	**0.14**
Exploration	Among-individual	−0.46, 0.50	-	0.01	0.02
	Within-individual	**0.37, 0.57**	-	**0.45**	**0.20**
	Phenotypic	**0.33, 0.53**	-	**0.41**	**0.19**
Novelty	Among-individual	−0.48, 0.51	−0.48, 0.50	-	−0.02
	Within-individual	**0.14, 0.39**	**0.34, 0.56**	-	0.06
	Phenotypic	**0.13, 0.36**	**0.30, 0.51**	-	0.05
Boldness	Among-individual	−0.48, 0.47	−0.76, 0.79	−0.45, 0.44	-
	Within-individual	**0.02, 0.28**	**0.07, 0.32**	−0.08, 0.20	-
	Phenotypic	**0.02, 0.27**	**0.07, 0.31**	−0.07, 0.19	-

**Table 5:** Results from LMM analyses with species, group, and acclimated temperature as fixed effects and ID as the random effect. The last column reports the marginal R^2^ with 95% credible intervals of the model (row: fixed effects) as well as the variance explained by species, group, acclimated temperature, and interaction.

Variables	Fixed Effects	Sum Sq	DenDF	F Value	*p* Value	Deviation Explained (%)
Sociability ^a^	species	13.8733	13.8733	167.6957	**˂0.001**	**18.60**
	group	1.5948	1.5948	19.2778	**˂0.001**	**6.880**
	acclimated temperature	7.0356	7.0356	85.0438	**˂0.001**	**17.34**
	species × group	0.7601	0.7601	9.1877	**0.003**	**22.10**
	Species × acclimated temperature	1.051	1.051	12.7043	**˂0.001**	**35.08**
Exploration ^b^	species	0.0096	157.33	0.1757	0.676	5.160
	group	0.19341	150.97	3.5389	0.062	10.49
	acclimated temperature	1.92932	281.49	35.3014	**˂0.001**	**32.89**
	species × group	0.24494	160.88	4.4817	**0.036**	**10.74**
	Species × acclimated temperature	0.63009	295.84	11.5289	**˂0.001**	**40.72**
Novelty ^c^	species	0.92054	147.64	11.2268	**0.001**	**10.86**
	group	0.48735	141.58	5.9437	**0.016**	**15.53**
	acclimated temperature	1.52759	272.37	18.6303	**˂0.001**	**27.80**
	species × group	0.14318	151.12	1.7462	0.188	19.33
	Species × acclimated temperature	0.0828	287.2	1.0098	0.316	26.49
Boldness ^d^	species	1.45385	386	1.3447	0.247	14.85
	group	0.67566	386	0.6249	0.430	25.74
	acclimated temperature	0.40093	386	0.3708	0.543	16.83
	species × group	1.18148	386	1.0928	0.297	29.70
	Species × acclimated temperature	0.56241	386	0.5202	0.471	12.87

Note: a: distance travelled near stimulus shoal. b, c: distance travelled. d: time spent continuously outside the shelter.

## Data Availability

The datasets generated and analyzed during the current study are available upon request from the corresponding author.

## Supplementary Materials

The following supporting information can be downloaded at https://www.mdpi.com/article/10.3390/ani14142101/s1.
