# Supplementary material for: Effect of Warming on Personality of Mosquitofish (Gambusia affinis) and Medaka Fish (Oryzias latipes)
Source: Animals (Basel). 2024 Jul 18;14(14):2101. doi: 10.3390/ani14142101 (PMC11273402; doi:10.3390/ani14142101)
Supplement: Supplementary file 1 [file animals-14-02101-s001.zip › animals-3091336-supplementary.pdf]

Supplement Material

Effect of Warming on Personality of Mosquitofish (*Gambusia affinis*) and Medaka Fish (*Oryzias latipes*)

By Rong Wang et al

Table

Table S1. Results of testing binary correlations between sociability (percentage of time spend near stimulus shoal), exploration (percentage of time spend moving), novelty (percentage of time spend moving) and bold-ness (time spent continuously outside the shelter) behaviors with MCMC general linear mixed effects models. The best estimates of correlation coefficients (values above the diagonal) and their 95% credibility intervals (values below the diagonal) are presented for among-individual, within-individual, and phenotypic correlations in *Gambusia affinis*. Significant results corresponding to correlation coefficients whose credibility intervals do not overlap zero are shown in bold.

		**Sociability**	**Exploration**	**Novelty**	**Boldness**
Sociability	Among-individual	-	0.10	0.05	-0.03
	Within-individual	-	0.03	0.03	0.01
	Phenotypic	-	0.02	0.03	0.01
Exploration	Among-individual	-0.63, 0.76	-	-0.03	0.23
	Within-individual	-0.20, 0.14	-	0.09	0.12
	Phenotypic	-0.16, 0.13	-	0.07	0.14
Novelty	Among-individual	-0.50, 0.66	-0.54, 0.49	-	0.08
	Within-individual	-0.13, 0.20	-0.08, 0.30	-	0.16
	Phenotypic	-0.12, 0.18	-0.09, 0.22	-	0.12
Boldness	Among-individual	-0.52, 0.48	-0.22, 0.61	-0.41, 0.56	-
	Within-individual	-0.15, 0.18	-0.07, 0.30	-0.33, 0.05	-
	Phenotypic	-0.13, 0.15	-0.01, 0.29	-0.27, 0.02	-

Table S2. Results of testing binary correlations between sociability (percentage of time spend near stimulus shoal), exploration (percentage of time spend moving), novelty (percentage of time spend moving) and bold-ness (time spent continuously outside the shelter) behaviors with MCMC general linear mixed effects models. The best estimates of correlation coefficients (values above the diagonal) and their 95% credibility intervals (values below the diagonal) are presented for among-individual, within-individual, and phenotypic correlations in *Oryzias latipes*. Significant results corresponding to correlation coefficients whose credibility intervals do not overlap zero are shown in bold.

		**Sociability**	**Exploration**	**Novelty**	**Boldness**
Sociability	Among-individual	-	0.04	-0.01	0.06
	Within-individual	-	0.07	0.12	0.11
	Phenotypic	-	0.06	0.11	0.10
Exploration	Among-individual	-0.53, 0.63	-	0.01	0.08
	Within-individual	-0.07, 0.20	-	0.004	**0.19**

	Phenotypic	-0.07,0.18	-	0.003	**0.18**
Novelty	Among-individual	-0.58, 0.52	-0.54, 0.55	-	0.13
	Within-individual	-0.26, 0.01	-0.14, 0.14	-	0.07
	Phenotypic	-0.24, 0.02	-0.13, 0.13	-	0.08
Boldness	Among-individual	-0.76, 0.78	-0.69, 0.79	-0.61, 0.75	-
	Within-individual	-0.03, 0.24	**0.06, 0.31**	-0.06, 0.21	-
	Phenotypic	-0.02, 0.23	**0.06, 0.31**	-0.06, 0.19	-

Table S3. Results from LMM analyses with species, group and acclimated temperature as fixed effects, and ID as the random effect. The last column reports the marginal R2 with 95% credible intervals of the model (row: fixed effects) as well as the variance explained by species, group, acclimated temperature and interaction.

Variables	Fixed effects	Sum Sq	DenDF	F value	*p* value	Deviation explained (%)
Sociability[a]	species	6.3035	152.04	70.7482	<**0.001**	**32.71**
	group	0.0101	145.35	0.1139	0.736	3.560
	acclimated temperature	0.0049	279	0.0553	0.814	7.440
	species × group	0.5058	155.47	5.6773	**0.018**	**36.27**
	Species ×acclimated temperature	0.6035	294.27	6.7731	**0.009**	**20.02**
Exploration[b]	species	0.021164	154.75	1.8882	0.171	8.430
	group	0.050604	148.89	4.5147	**0.035**	**20.51**
	acclimated temperature	0.189332	276.56	16.8915	<**0.001**	**25.56**
	species × group	0.039127	158.26	3.4907	0.064	19.24
	Species ×acclimated temperature	0.037283	290.34	3.3263	0.069	26.26
Novelty[c]	species	0.000035	168.99	0.0133	0.909	2.530
	group	0.000441	161.91	0.1677	0.683	1.010
	acclimated temperature	0.042048	293.05	15.999	<**0.001**	**56.96**
	species × group	0.000484	172.48	0.1841	0.668	3.290
	Species ×acclimated temperature	0.000099	307.11	0.0378	0.846	36.20
Boldness[d]	species	1.45385	386	1.3447	0.247	14.85
	group	0.67566	386	0.6249	0.430	25.74
	acclimated temperature	0.40093	386	0.3708	0.543	16.83
	species × group	1.18148	386	1.0928	0.297	29.70
	Species ×acclimated temperature	0.56241	386	0.5202	0.471	12.87

Note: a: percentage of time spend near stimulus shoal. b, c: percentage of time spend moving. d: time spent continuously outside the shelter.

Supplementary Figure

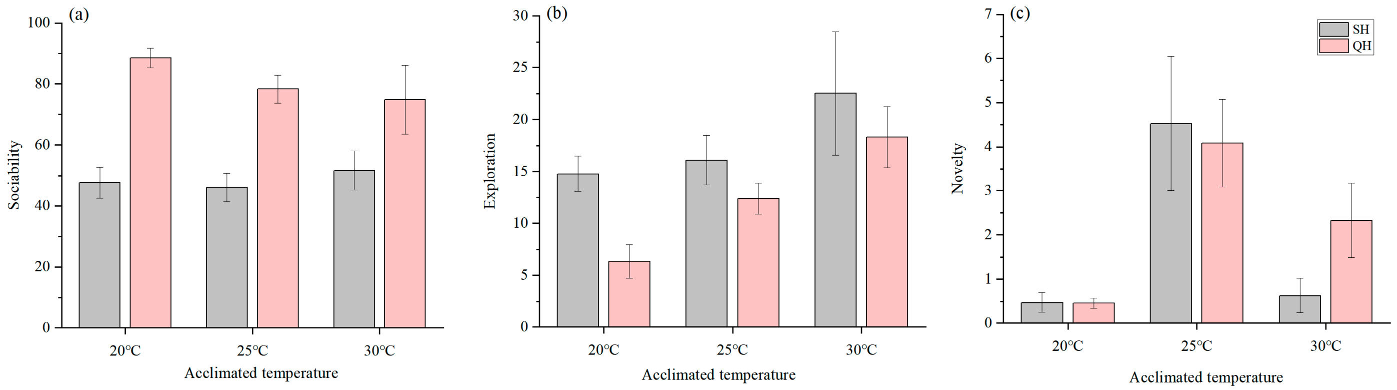

Figure S1: The effect of temperature changes in the high temperature group on the personality of animals. (a) sociability: percentage of time spend near stimulus shoal, (b) exploration: percentage of time spend moving, (c) novelty: percentage of time spend moving. Group SH and SL represents mosquitofish exposed to warming and control groups, respectively. Group QH and QL represents medaka fish exposed to warming and control groups, respectively.
